# Fabrication and Characterization of Flexible and Miniaturized Humidity Sensors Using Screen-Printed TiO_2_ Nanoparticles as Sensitive Layer

**DOI:** 10.3390/s17081854

**Published:** 2017-08-11

**Authors:** Georges Dubourg, Apostolos Segkos, Jaroslav Katona, Marko Radović, Slavica Savić, Georgios Niarchos, Christos Tsamis, Vesna Crnojević-Bengin

**Affiliations:** 1 Nano and Microelectronics Group, BioSense Institute, 21000 Novi Sad, Serbia; marrad@biosense.rs (M.R.); slavicas@biosense.rs (S.S.); niarchos@uns.ac.rs (G.N.); bengin@uns.ac.rs (V.C.-B.); 2 Institute of Nanoscience and Nanotechnology, NCSR “Demokritos”, Patriarhou Gregoriou & Neapoleos, 15310 Aghia Paraskevi, Greece; a.segkos@inn.demokritos.gr (A.S.); c.tsamis@inn.demokritos.gr (C.T.); 3 Faculty of Technology, University of Novi Sad, 21000 Novi Sad, Serbia; jkatona@uns.ac.rs

**Keywords:** humidity sensors, flexible substrate, TiO_2_ nanoparticles, screen-printing, laser ablation

## Abstract

This paper describes the fabrication and the characterization of an original example of a miniaturized resistive-type humidity sensor, printed on flexible substrate in a large-scale manner. The fabrication process involves laser ablation for the design of interdigitated electrodes on PET (Poly-Ethylene Terephthalate) substrate and a screen-printing process for the deposition of the sensitive material, which is based on TiO_2_ nanoparticles. The laser ablation process was carefully optimized to obtain micro-scale and well-resolved electrodes on PET substrate. A functional paste based on cellulose was prepared in order to allow the precise screen-printing of the TiO_2_ nanoparticles as sensing material on the top of the electrodes. The current against voltage (I–V) characteristic of the sensor showed good linearity and potential for low-power operation. The results of a humidity-sensing investigation and mechanical testing showed that the fabricated miniaturized sensors have excellent mechanical stability, sensing characteristics, good repeatability, and relatively fast response/recovery times operating at room temperature.

## 1. Introduction

Humidity sensors are employed today in a wide range of applications, including environmental monitoring, automotive, industrial process, healthcare, agriculture, and increasing indoor air quality in smart buildings. Several kinds of humidity sensors are available based on different transduction principles, such as resistive, capacitive, optical, and surface acoustic wave [1,2,3,4]. However, resistive-type sensors have the advantage to be cheaper and easier to read out over the other ones.

Typically, rigid substrates like ceramic, glass, or silicon are used as the fundamental building blocks of humidity sensors; but, recent advancements in the field of printed electronics show increased potential for the substitution of rigid substrates by flexible ones, since the latter potentially reduce the cost of sensors and offer good mechanical flexibility. Examples of flexible sensors integrating additional electronic functions like readout electronics [5,6], thermal compensation systems [7], and other sensors [8,9] have opened a new route towards multi-functional sensors fabricated on flexible substrate. Despite that, silicon technology is still attractive for the fabrication of sensors due to its mass-production capability, its high degree of miniaturization resulting in high integration density, and, consequently its considerable cost reduction for sensor devices [10,11]. Indeed, as given in the paper of Moore in 1965 [12]: “With unit cost falling as the number of components per circuit rises”, the cost of one sensor must also decrease as more sensors are put on the substrate.

In addition, due to their very small size, silicon-based devices can be integrated with a flexible substrate. For example, a silicon strain sensor and multiplexed silicon non-volatile memory were transferred onto flexible substrate for wearable electronics [13,14]. Miniaturization is then an important issue in printed electronics, which needs to be assessed to consider it a valuable alternative to silicon technology.

In the case of resistive-type sensors, the size of a device depends mainly on the surface area covered by the electrodes. Low-cost printing technologies such as ink-jet or screen-printing are forecasted to dominate the printed electronics era, since they allow high-volume production [15,16]. However, for electrode designs, the line resolution/width achievable by printing technologies cannot reach micro-scale features generally higher than 100 μm, resulting in a large surface area. Thus, alternative technologies need to be considered to obtain miniaturized devices. Photolithography coming from expensive CMOS (Complementary Metal–Oxide–Semiconductor) technologies allows the design of micro-scale electrodes with high resolution in a large-scale manner, and this technology can be also employed for the mass production fabrication of sensors on flexible substrate [17,18,19,20]. Nevertheless, chemical and baking steps are required in photolithography, which limits it to chemically resistant substrates such as polyimide.

Laser technology is gaining interest as another alternative micropatterning technique due to its high precision and the possibility to use it in open air without clean room facilities. This process was used for the fabrication of ozone sensors, and was compared with photolithography in [21]. It was shown that this method can reach features of up to 60 μm. However, smaller features should be obtained to enhance its potential for sensor fabrication.

Furthermore, to take advantage of miniaturization, it is important to select an adequate sensing material. Among the various sensing materials, metal oxide nanomaterials possess good properties such as chemical and physical stability and high mechanical strength, and they have a high surface-to-volume ratio that makes them a perfect candidate for sensor applications. In recent years, TiO_2_ has received wide attention and has found applications in many promising areas, such as photovoltaics, photocatalysis, and sensors [22,23,24,25,26]. Many examples of humidity sensors based on TiO_2_ can be found in the literature [27,28,29,30]. The ability of the sensing material to be integrated in industrial production depends on the fabrication route used for its deposition and patterning. In the case of humidity sensors, the most standard methods to deposit TiO_2_ are the spin-coating, dip coating, or microdroper processes [27,28,29,30,31], which are suitable for prototypes, but cannot be considered for large-scale process. In fabrication route selection, several factors must be considered, such as costs, throughput, and reproducibility, and the procedure ought to be compatible with the substrate, especially in terms of temperature. The screen-printing process meets all of the above, which has been demonstrated in [32,33], where the screen-printing of TiO_2_ nanomaterials was used for dye-sensitized solar cells and electrodes.

In this context, the aim of this work was to introduce a cost-efficient and low-temperature procedure that allows the large scale fabrication of humidity sensors on flexible PET(Poly-Ethylene Terephthalate) substrate. The undertaken multidisciplinary approach combines expertise in materials science and chemistry, and fabrication processes and sensor characterization, aiming to present comprehensive bottom-up research in the field of flexible electronics and sensors. One of the main goals of the conducted research is not only to introduce an innovative technology process for the fabrication of sensor devices, but also to provide a proof-of-concept through extensive mechanical testing and a humidity response characterization of the fabricated miniaturized sensors.

## 2. Materials and Methods

### 2.1. Fabrication

**Figure 1:**
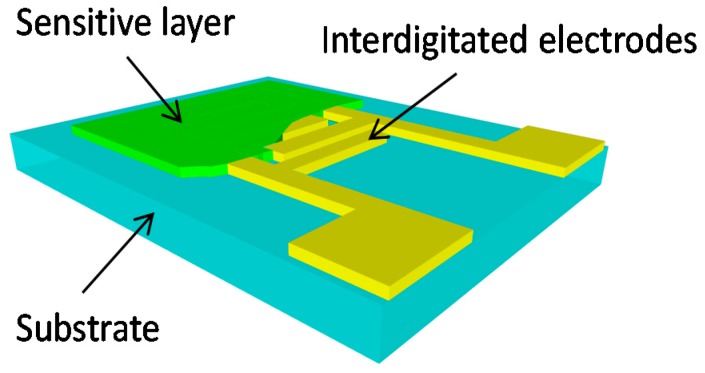
Schematic model of resistive-type chemical sensor.

The concept of the sensor is based on the resistive transduction principle, which consists in the deposition of a TiO_2_-based sensitive layer on interdigitated electrodes (IDE) previously patterned on a flexible substrate (Figure 1). PET substrate was chosen as a flexible building block for the sensor design, because this material is biodegradable, cost effective, and widely available. The underlying principle of the sensor’s operation lies in fact that the absorption of water molecules by the sensitive film results in an increase of the film’s electrical conductance. The conductance change as function of the humidity level can be easily quantified by measuring the resistance between the interdigitated electrodes.

Keeping the intended final application closely in mind, we aimed to develop a simple and economic technological process for the fabrication of humidity sensors, in order to be able to preserve their attractiveness as low-cost, potentially mass-produced devices. The fabrication process of the sensors proposed in this work is fast, compatible with roll-to-roll technologies, and does not require the high-cost of the semiconductor manufacturing equipment and high temperature steps normally used for silicon or ceramic fabrication.

**Figure 2:**
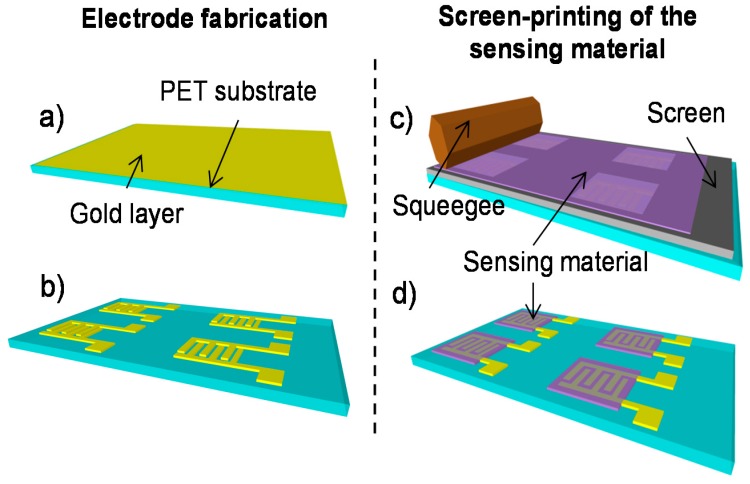
Process sequence of the humidity sensors: (**a**) Deposition of the gold layer on PET (Poly-Ethylene Terephthalate) substrate; (**b**) Laser ablation of the gold layer; (**c**) Screen-printing of the TiO_2_ nanoparticles; (**d**) Sensors after screen-printing.

The process sequence for the fabrication of the flexible humidity sensors is schematically illustrated in Figure 2. Initially, a gold layer was deposited by electron beam evaporation on a commercial PET substrate (Figure 2a). Afterwards, the resulting layer was directly patterned by laser ablation using a short pulse laser (Nd:YAG-1064 nm, Rofin) in order to create micro-scale interdigitated electrodes (Figure 2b). The next step is the patterning of the sensitive layer on top of the IDE in a low-cost manner. For this purpose, a TiO_2_-based paste was prepared and then screen-printed (Figure 2c) in order to cover locally the surface of the electrodes (Figure 2d).

The following sections describe the process sequences in more detail.

#### 2.1.1. Laser Ablation of the Interdigitated Electrodes

The deposited gold layer was patterned by laser ablation (Nd:YAG-1064 nm, Rofin, Plymouth, MI, USA) for the design of micro-scale interdigitated electrodes. This powerful technique used for the micromachining of microdevices consists of the creation of an effective heat zone by a focused laser beam, which induces a localized physical state transition allowing the ablation materials [34]. In order to obtain micro-scale and well-resolved electrodes, the laser ablation process was optimized as proposed in [35,36]. First, the pulse overlapping was adjusted by using the maximum available frequency of 65 kHz and a low raster speed of 80 mm/s in order to achieve a continuous ablation line without damaging the substrate by thermal accumulation. Next, to obtain the micro-scale features, the laser ablation process was optimized by modification of the current values. A current of 23 A was found to be an ideal value to succeed with the complete and selective ablation of the metal layer at the micro-scale. Using these parameters, the thin layer of gold was patterned without damaging the PET substrate.

#### 2.1.2. Sensitive Layer: Preparation and Screen-Printing

A screen printing process, which is a cost-effective, time-saving, and mass-production fabrication process, was used for the deposition and patterning of the sensing material. This technique consists in using a squeegee for depositing a paste through a screen stencil, which allows for the direct patterning of functional pastes on a large variety of substrates [37,38].

In this work, a TiO_2_ nanoparticles-based paste was used for the fabrication of the sensitive layer, and was developed specially for the screen-printing process. Water was chosen as the main solvent of the paste because it is readily available and environmentally friendly. First, a 2.5 wt % hydroxypropylmethyl cellulose (HPMC, Methocel^®^ K15, Colorcon, Dartford, UK) was dissolved in water. Next, a 6.1/1 wt % propylene glycol/n-propanol mixture was prepared, where both the propylene glycol and n-propanol were of p.a. quality obtained from Kemika Zagreb, Croatia. Then, a 2.7 wt % solution of a dispersant (Solsperse 40000, Lubrizol, Wickliffe, OH, USA) in the propylene glycol/n-propanol mixture was prepared. The HPMC solution and the solspers solution were mixed at 1:1 wt ratio by means of an IKA RW20 overhead stirrer for 10 min at 1500 rpm. TiO_2_ powder (anatase, Sigma Aldrich, St. Louis, MO, USA) was dispersed (IKA RW20, 1500 rpm for 20 min) in the mixture to obtain a 7.5 wt % dispersion of TiO_2_. The screen-printing of the TiO_2_ paste was performed using a semi-automatized screen-printer (EKRA 2H screen-printer, Dornstadt, Germany), which is a widespread industrially-applied piece of equipment. A screen was fabricated using a 30 μm thick photopolymer film (Koenen, Ottobrunn-Riemerling, Germany). The mesh used for the screen-printing of the TiO_2_ paste onto the surface of the electrodes was characterized by a wire diameter of 30 μm. Finally, the samples were kept at room temperature for 2 days to attain complete dryness.

### 2.2. Measurements

The precision patterning of the devices and the morphology of the screen-printed TiO_2_ film were examined by scanning electron microscope (HITACHI TM3030) and atomic force microscopy (AFM), whose images were taken with an NTEGRA prima microscope in semi-contact mode. The composition of the TiO_2_ was investigated by Energy Dispersive X-Ray Analysis (Bruker XFlash), and the electrical characterization was performed using a Yokogawa–Hewlett-Packard semiconductor probe analyzer.

The humidity sensing properties of the fabricated sensors were investigated at room temperature (25 °C) by using an indigenously custom-designed humidity setup as described in [36]. The sensor to be analyzed was placed inside a sealed Teflon chamber, along with a reference sensor (Hanna instrument) that was used to monitor in real time the temperature and relative humidity (RH) inside the chamber. The water vapors were generated by driving gases (N_2_ and O_2_) inside a sealed bubbler containing water, and the resulting vapors were then carried to the experimental chamber where the sensors were tested. The humidity level inside the chamber was controlled by adjusting the concentration of the driving gases with mass-flow controllers and flow meters (Brooks Instruments). The humidity sensing response was recorded through the change in resistance caused by varying the RH. A Keithley multimeter, driven by a custom-designed Labview-based interface, was used for monitoring in real time the resistance across the IDEs.

## 3. Results and Discussion

**Figure 3:**
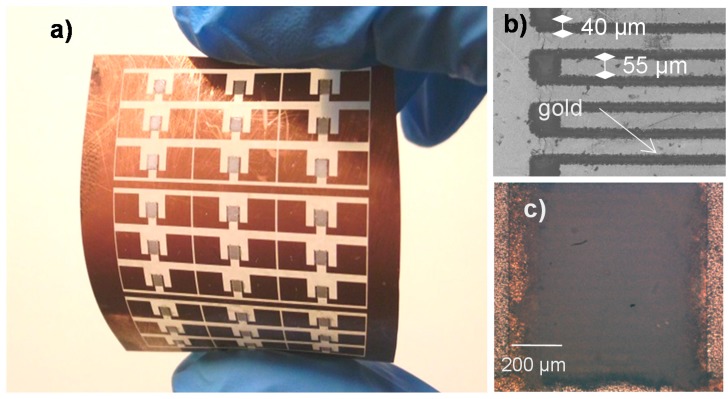
(**a**) Picture of 3 × 3 sensor matrices fabricated on PET substrate; (**b**) SEM image of the interdigitated electrodes; (**c**) Optical image of the humidity sensors.

Using the process described above, the large-scale fabrication of humidity sensors has been successfully achieved. Figure 3a shows matrices of 3 × 3 sensors printed on PET substrate. Figure 3b depicts an SEM image of the ablated interdigitated electrodes on the PET substrate. This image indicates that the surfaces of the electrodes’ structures subjected to pulse ablation are highly consistent and spatially well-resolved. An individual digit of an electrode is 700 μm long and 55 μm wide, and it is separated by a gap of 40 μm to the next digit. Here, small electrode geometry was obtained compared to the standard printed sensors, which are generally above 100 μm.

Figure 3c shows an optical picture of the interdigitated electrodes covered by the screen-printed TiO_2_ film. The resulting TiO_2_ film is well aligned with the film, covering perfectly the surface of the electrodes. This perfect alignment of the screen-printed film is also confirmed at larger scale, as shown in Figure 3a. The screen-printed TiO_2_ nanoparticle-based film, defining the active area of the humidity sensor, forms a rectangle of 1 mm width and 1.5 mm length (Figure 3b).

**Figure 4:**
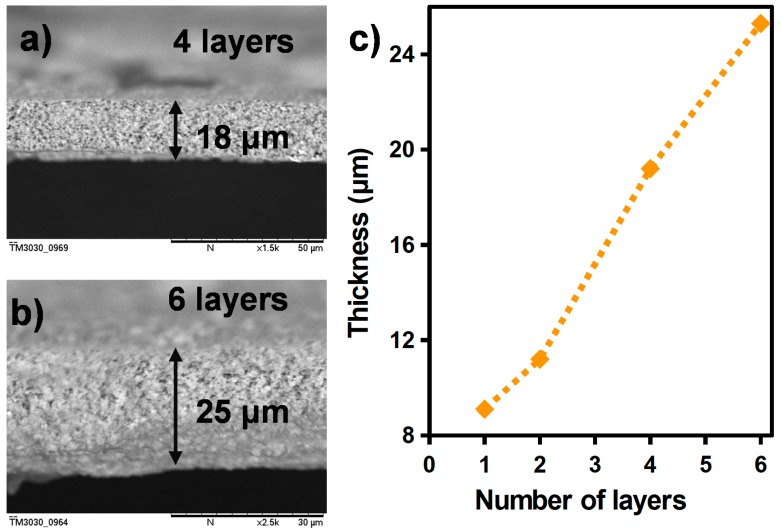
SEM image of a cross-section of the TiO_2_ film (**a**) for four printed layers and (**b**) for six printed layers; (**c**) thickness of the final TiO_2_ film as function of the number of printed layers.

The thickness of the TiO_2_ can be controlled by the number of printed layers. Indeed, Figure 4a,b represents a cross-section of the TiO_2_ film after the printing of four layers and six layers, where the thicknesses were measured to be approximately 18 μm and 25 μm, respectively. The evolution of the thickness as function of the number of printed layers is summarized in Figure 4c.

**Figure 5:**
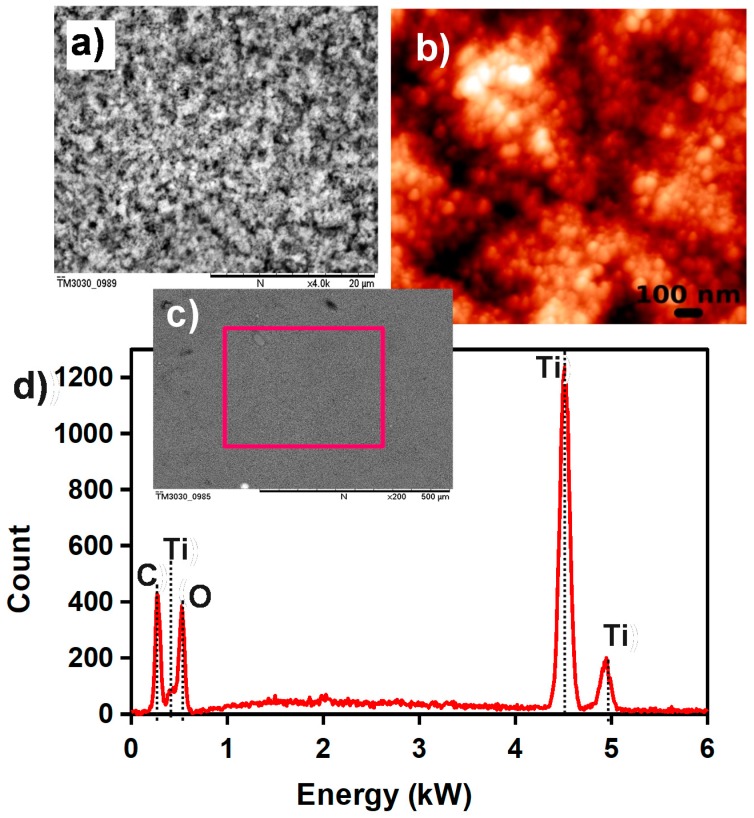
(**a**) SEM image of the screen-printed TiO_2_ film; (**b**) AFM image of printed TiO_2_ nanoparticles; (**c**) large area SEM image of the TiO_2_ film; and (**d**) EDX spectrum of the selected area on TiO_2_ film shown in (**c**).

The sensing properties are based on the change in the electrical conductance of the sensitive layer with the adsorbed water, which depends on the surface characteristics of the film. The surface morphology of the TiO_2_ film was investigated using SEM and AFM techniques. Figure 5a shows the SEM image of a TiO_2_ layer with high magnification, where the porous structure of the TiO_2_ film can be observed, which is favorable for water vapor absorption due to the large surface area [39]. Figure 5b shows an AFM image of the TiO_2_ film, where the spherical structure of the TiO_2_ nanoparticles with a grain size of less than 100 nm, and the porosity of the film, can be clearly observed. Also, we can see in Figure 5d that the film formed by TiO_2_ after screen-printing is quite uniform and homogeneous along the sensor. Next, an energy dispersive X-ray spectrometer (EDX) was employed to study the structural composition of the printed titanium dioxide film. Figure 5c shows the EDX spectrum of the selected area shown in Figure 5d, where the main peaks correspond to titanium and oxygen, indicating that the surface is well covered with TiO_2_. The presence of carbon can be clearly observed, and it has been attributed to organic components of the functional paste. Note that, among them, an important component is the binder (hydroxypropylmethyl cellulose), since it assures a strong binding between TiO_2_ nanoparticles and a good adhesion of the TiO_2_ film with the substrate, improving the stability of the TiO_2_ film.

**Figure 6:**
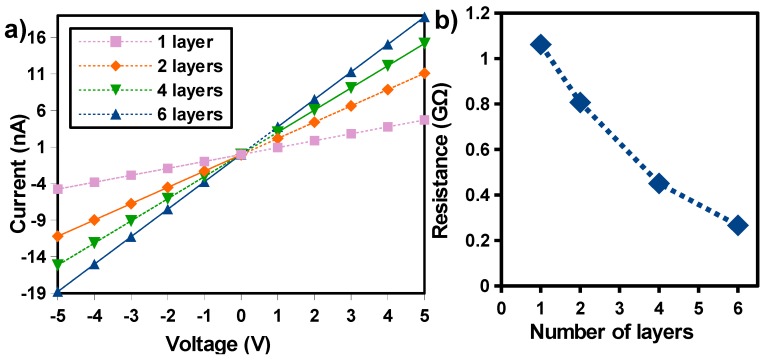
Current against voltage (I–V) characteristics of (**a**) the devices with different numbers of printed TiO_2_ layers; (**b**) resistance of the TiO_2_ as function of the number of printed layers.

TiO_2_ film should possess good electrical performances to allow for precise and stable resistance measurements. Then, the electrical characteristics of the printed structures should be investigated as well. Current against voltage (I–V) measurements were obtained on sensors printed with one, two, four, and six layers by sweeping the applied voltage from −5 to 5 V. A typical current reading, as shown in Figure 6a, clearly demonstrates that the TiO_2_ film provides a connecting Ohmic electrical contact between pairs of Au electrodes with constant resistance over the supply voltages. That means that a low voltage operation does not hinder the sensitivity, which is essential for low power operation. On the other hand, the conductance of the printed layer should be high enough to be measurable without a high-precision instrument. Figure 6a highlights the influence of the number of printed layers on the electrical performance of the film. For one printed layer, the variation in current is about 4 nA at 5 V bias, which reveals a poor conductance of the TiO_2_ film. Generally, post-processing steps such as annealing are required to improve the conductivity of the material, leading to an increase in energy consumption and producing additional cost. In this work, in order to develop a low cost and low-temperature process adapted to flexible substrates, we have formulated a recipe for a functional paste that can be used for the printing of several TiO_2_ layers, and that can preserve the original material physical and transport properties. Indeed, in Figure 6a, we can see that the sensor current increased as the number of successive printed layers is increased, due to the added TiO_2_ nanopartices (NPs). This leads to a drop of resistance from about 1 GΩ to 266 MΩ (Figure 6b). With six printed layers, the resulting resistance (266 MΩ) is low enough to make the sensor compatible with a simple and low-powered electronic scheme, such as a Wheatstone bridge, for the signal read-out.

Afterwards, humidity sensing performance was evaluated using the following equation to define the sensors’ response:

$$S\left(\%\right)=\frac{R_{\mathrm{ini}}-R_{\mathrm{mes}}}{R_{\mathrm{ini}}}\times 100$$

where R_mes_ are the resistances at a given humidity level, and R_ini_ is the resistance at zero humidity used as a baseline.

**Figure 7:**
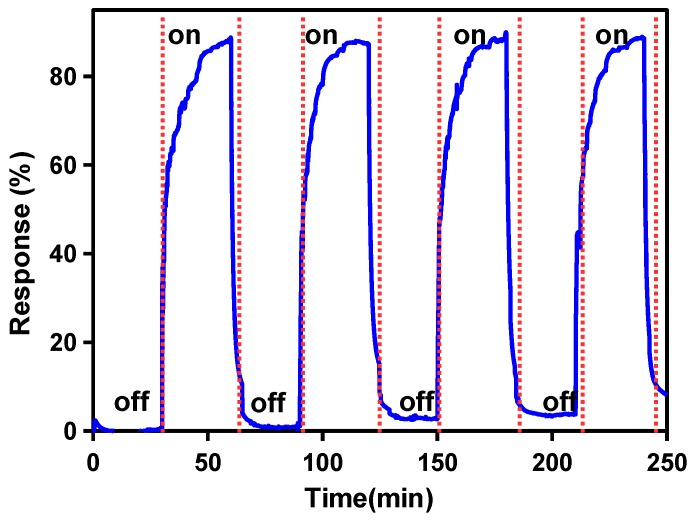
Sensor response under dynamic cycles between 0% and 72% relative humidity (RH), at 25 °C.

Reproducibility is one of the first requirements for a sensor’s application. Typically, it is defined as a condition wherein the sensors exhibit multiple vapor adsorptions/desorption behaviors under cyclic operating conditions. In order to examine this, the humidity environments of the sensor were sequentially changed from 0 to 70% in periods of 30 min for several sorption and desorption processes. Figure 7 reveals that during the fourth response/recovery cycles, the sensor response shows a good sensing repeatability during cycling tests, which represents another advantage for its potential application. However, a drift of about 8% in the initial value of the response can be observed in Figure 7. This was attributed to residual moisture that had accumulated in the TiO_2_ film after several sorption and desorption processes. Indeed, the highly porous structure of the TiO_2_ film highlighted in Figure 5a,b can easily trap moisture, producing the observed drift in the measurements.

Next, in order to study further the characteristics of our humidity sensors, it is important to investigate the sensors’ response at different humidity levels.

**Figure 8:**
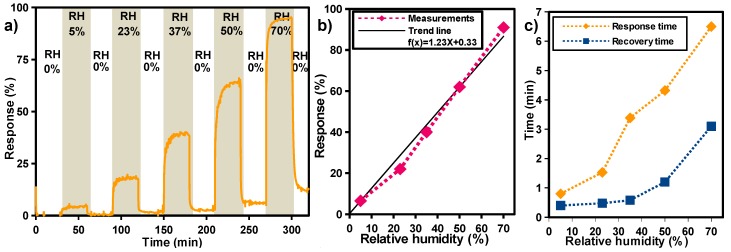
(**a**) response curve of the sensor to gradually increased humidity levels, ranging from 0 up to 72%; (**b**) sensor response as function of the relative humidity; (**c**) response and recovery time of the sensor as function of the relative humidity.

Figure 8a shows the sensors’ response for several dynamic cycles of absorption/desorption at humidity levels varying from 0 to 70%. It is important to mention that low relative humidity levels were detected with designed miniaturized sensors, introducing a significant improvement in comparison to the other flexible humidity sensors found in the literature [40,41,42,43]. This can be attributed to the highly porous surface of the printed TiO_2_ film, which results in a large surface area providing more surface active sites and paths for water molecule adsorption and diffusion [39].

Figure 8b presents the sensors’ response as a function of humidity level, where it can be observed that sensor response is linearly proportional to the relative humidity level, implying a more precise measurement at a low humidity level and simple calibration, which are important parameters for potential sensor application.

The response and recovery times are also very important factors to determine the performance of humidity sensors, and they also need to be evaluated. The response time is the time taken by a sensor to achieve 90% of the maximum response, and the recovery time is the time needed for the senor to drop to 10% of its initial response. Both parameters were calculated from a long cycle time (30 min), which was used to ensure that the device response reached its saturated limit without any noticeable drift.

Figure 8c shows the response and recovery time as function of the relative humidity level including the equilibration time of water vapor inside the test chamber. In this Figure, it can be seen that the response and the recovery times are fast in a range from 5 to 40 % RH, varying between 40 s and 3 min for the response times, and about 50 s concerning the recovery times. However, the response and recovery times become much slower at higher RH levels (>50% RH). This could be attributed to the humidity sensing mechanism. In fact, at low RH, the decrease of resistance is mainly due to the chemisorbtion of water molecules by the active sites available on the TiO_2_ surface. In that case, the dominant charge transport mechanism is electronic transport, which is much faster than proton conduction. On the other hand, the subsequent layer of the water molecule is generally physisorbed by double hydrogen bonding with the hydroxyl groups formed on the previous water layer [30,39]. Afterwards, successive physisorbed water layers are accumulated on the surface of the TiO_2_ film as the humidity level increases. In that case, the proton conduction mechanism becomes dominant, which could explain the slower response times for high humidity levels.

**Figure 9:**
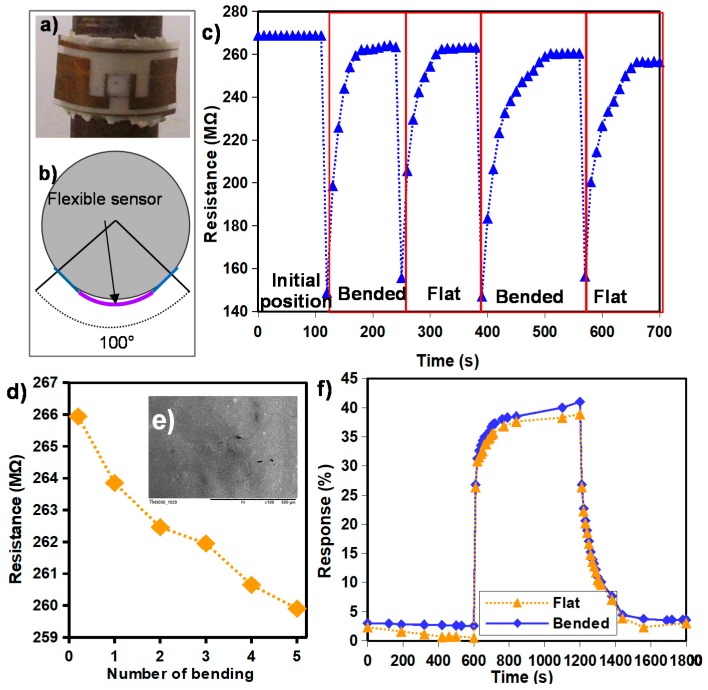
(**a**) picture of the sensor placed on cylinder; (**b**) schematic of the bended sensor; (**c**) resistance under dynamic bending cycles; (**d**) resistance value as function of the bending cycles; (**e**) SEM picture of the TiO_2_ after bending cycles; (**f**) response curves of the sensor to 35% RH when tested in a flat and a bended position.

Mechanical stability is essential to flexible electronic devices, especially for applications where high stability over the mechanical deformation is required, such as wearable electronic and smart food packaging. Therefore, the influence of the mechanical strain on the electrical behavior of the devices has to be explored. To do so, bending experiments were performed by attaching the flexible sensors to a cylinder (Figure 9a) and the curvature angle was calculated to be approximately 100°, as depicted in Figure 9b.

Figure 9c shows the resistance change during several bending and return to flat position cycles in periods of 5 min. It can be seen that the resistance decreased during the bending experiments, but it retrieved its initial value quickly after the mechanical excitation, i.e., after a relaxation time of about 1 min.

Figure 9c exhibits the resistance as a function of the number of bending cycles. The device showed only a slight decrease in resistance (2.3% of the initial value) after five cycles. Moreover, a scanning electron microscopy (SEM) analysis revealed no morphology change of the film caused by mechanical bending (Figure 9d).

To validate the stable sensing operation under mechanical deformation, humidity measurements were performed when the sensor was in a flat position and bended at 100°.

At each indicated position, the sensor was exposed to RH varying from 0 to 35% RH in periods of 10 min. Note that the measurements in a bended position were performed 2 min after bending the sensor in order to leave it enough time to recover its initial resistance value. Figure 9e shows that the response of the sensor when it was bended increased by less than 3% from that measured when it was in a flat position. It can be concluded that the sensors’ response showed negligible effect over the mechanical strain.

The obtained results indicate that the TiO_2_ paste formulation offers high mechanical stability for a TiO_2_-sensitive layer when it is printed on a plastic substrate, which consequently allows the devices to be used for flexible sensor applications.

## 4. Conclusions

An original and innovative process for the large-scale production of flexible and miniaturized humidity sensors with TiO_2_ nanoparticles as sensing material was proposed. This method results from the association of two different approaches: laser ablation and screen-printing. The first approach is coherent for the patterning of micro-scale interdigitated electrodes. The second one is particularly adapted for the industrial integration of metal-oxide-based sensitive film on flexible substrate. Both approaches are fast, cost-effective, and do not require annealing and chemical treatment, which makes them compatible with any kind of flexible substrates. The electrical measurements of the investigated sensors revealed Ohmic behavior, and the electrical properties of the devices were improved by printing successive layers. Mechanical testing showed very good stability of the electrical properties and humidity response of the investigated sensors. The humidity sensing properties were evaluated by the measurement of resistance change with variation in the humidity. The linear response of the fabricated sensitive layer, in range from 5 to 70% relative humidity, reveals great potential for environmental monitoring and humidity sensing applications. In addition, the sensors showed good repeatability and a relatively fast response time. Therefore, the possibility to fabricate miniaturized sensors in a large-scale manner, with preserved good sensing properties, paves the way to low-cost solutions for sensor technologies printed on flexible substrates.
